# Socio-Organizational Dimensions: The Key to Advancing the Shared Care Record Agenda in Health and Social Care

**DOI:** 10.2196/38310

**Published:** 2023-01-26

**Authors:** Kathrin Cresswell, Stuart Anderson, Hajar Mozaffar, Andrey Elizondo, Marcia Geiger, Robin Williams

**Affiliations:** 1 Usher Institute The University of Edinburgh Edinburgh United Kingdom; 2 School of Informatics The University of Edinburgh Edinburgh United Kingdom; 3 Business School The University of Edinburgh Edinburgh United Kingdom; 4 Institute for the Study of Science, Technology and Innovation The University of Edinburgh Edinburgh United Kingdom

**Keywords:** integrated care, sociotechnical, socio-organizational, shared care records, health care, healthcare, social care, digital health

## Abstract

Integrating health and social care delivery with the help of digital technologies is a grand challenge. We argue that previous attempts have largely failed to achieve their objectives because implementers and decision makers disregard the complex socio-organizational dimensions of change associated with initiatives. These include structural and organizational complexity inhibiting the development of shared care pathways; professional jurisdictions, interests, and expertise; and existing data and governance structures. We provide an overview of those dimensions that can inform strategic decisions going forward, thereby contributing to the chances of success of shared care initiatives.

## Introduction

Drivers for digitally supporting the integration of the health and social care sectors include visions of improved patient experience and engagement, personalized care, improved patient safety, reduced cost, and increased availability of data for service planning and research [1]. However, despite some successes [2], efforts to create shared care records across health and social care settings have to date been largely unsuccessful, particularly at scale [3]. A key underlying reason is the limited attention among implementers and strategic decision makers to the interplay of technological and socio-organizational dimensions of change. Increasing consideration of these factors is crucial going forward to enhance the prospects of success and minimize patient risks and disruption of care delivery. Here, we summarize key technological and socio-organizational considerations.

## Technological and Socio-Organizational Considerations That Can Support the Shared Care Agenda

To deliver integrated health and social care services, diverse organizations and professional groups with differing needs and practices must share information. However, this information resides on a historical accumulation of separately developed systems, implemented on different proprietary platforms with limited interoperability to support the particular activities of various stakeholders. Harmonization is problematic, as the differing informational needs of organizations and groups are difficult to integrate into a single pathway of information flow [4]. Additionally, this may involve disrupting existing information flows that are embedded in current practices. For example, accident and emergency departments may require a general picture of the patient; visiting nurses and after-hours consultants may need to have access to primary care and secondary care information; hospital consultants may require a comprehensive understanding of a particular condition; primary care practices and social workers need to monitor and support patient health and well-being through engagement with various health and care services; and biomedical researchers and health service planners may seek to promote data linkage across large populations. The design of integrated information architectures should start by considering these diverse requirements in different contexts and roles, in terms of who needs to share what kinds of data, in what quantities, when, at what speed, and to achieve what objective. Otherwise, efforts to promote the digitally enabled integration of health and social care are likely to fail, with new functionalities being only partly utilized by various stakeholder groups, and not being incorporated into routine practices [5,6]. Unfortunately, integrated information architectures are never designed on a “green-field site,” so new architectures need to make provision to incorporate some pre-existing elements.

Thus, there is now a need to move toward a more holistic view of change to advance the shared care agenda. This should involve developers not only taking into account technological dimensions of change (eg, harmonizing standards, systems, and architectures) but also the institutional transformations necessary to promote shared care pathways across organizational and professional domains with varying types and levels of complexity and needs. Such socio-organizational aspects relate to existing organizational and structural differences across health and social care settings, as well as setups and practices that in some cases inhibit data sharing and shared workflows. For example, professional and organizational jurisdictions, interests, governance arrangements, concerns about losing control of the data, and, in some instances, competition between providers may result in a reluctance to share data. Awareness of some of these issues is increasing, with some work giving consideration to normative integration across professionals [7]. Silos may inadvertently be reinforced by existing organizational and technological structures, including existing infrastructures and legacy systems that are simply not designed for sharing data across settings that often vary significantly in relation to digital maturity. For example, electronic record systems designed for acute care providers do not cater well for community, mental health, and social care.

Table 1 summarizes such technological and socio-organizational considerations, which we hope will help planners and implementers consider the range of dimensions required to tackle this grand challenge. Although we focus here on the perspective of single organizations, it is important to recognize that these developments are situated within and shaped by the wider context. Solutions that work in one setting may struggle elsewhere due to differences in health service organization, funding, and regulation [8]. For example, social care is much more varied in terms of services available than health care (including the public, private, and voluntary sectors).

**Table 1:** Socio-organizational challenges associated with the digitally enabled integration of care.

Dimension		Description
**Socio-organizational dimensions**		
	Structural complexity [9,10]	Health and social care delivery includes a large number of organizations that vary in size from single individuals to extremely large organizations. The organization is ad hoc and decentralized. Current structures are not set up for integrated and shared work.
	Definition of shared care pathways [11]	Shared care pathways across organizations are poorly defined, resulting in a lack of integrated vision that could help with the alignment of stakeholders. A key challenge is to establish technical and organizational methods to develop dependable new workflows and pathways that cut across organizational and professional boundaries.
	Organizational complexity [12]	Care organizations are situated within multiple levels of administrative structures, all of which have different incentives and expectations around the idea of sharing data. National or regional reporting requirements are not necessarily aligned with the informational needs of particular organizations and professionals. Powerful visions and nostrums like “seamless data flow” possibly conflict and divert attention from these different reporting requirements. Different actors attach diverse understandings and meanings to data and various settings have different ways to depict and view information.
	Professional jurisdictions, interests, and expertise [13]	Some professions and individuals are reluctant to share data if this may be perceived as a threat to professional autonomy.
	Data ownership [14]	There may be a lack of clarity regarding who owns the data held in various systems and shared across settings.
	System configuration [15]	Functionality developed or configured in the context of a particular setting has a tight fit to the needs of that setting. This means that this functionality may have difficulty fulfilling the needs of other settings.
	Incentives [16]	Different organizations and professions have diverse incentives and disincentives for data sharing.
	Data overload [9,10,17]	There is a tension between sharing data widely and being able to access data relevant to the unique setting or profession. This may result in potential issues with data overload, and problems surrounding discrimination or action.
	Vision [18]	The models of data sharing are not well articulated, and there is no clear vision of how to make the transition toward integrated health and social care infrastructures.
	Information governance [19]	Models of data sharing open up the potential for new governance models, but these are not clearly articulated. There may be information governance concerns, particularly when data leaves the health domain (eg, types of consent and levels of access).
	Liability [20]	There may be a fear of liability among health care staff if many other stakeholders can see their records. This may have unintended consequences (eg, not recording important information).
	Skills [21]	There is a lack of interoperability skills and data skills to facilitate digitally enabled changes.
	Training [22]	Shared care pathways and relationships are not included in health and social care work training.
	Governance structures [23]	Different architectures can be linked with different structures of governance. However, it can be difficult to know beforehand which technology will be developed or procured and what the governance implications are. Timescales for achieving infrastructural change also contrast with short policy and funding cycles.
	Distributed knowledge [24]	Shared care records imply a logical compilation of complex knowledge that is distributed across dispersed arrays of actors. However, this knowledge is often not logically organized.
	Social determinants of health [25]	Shared care record initiatives need to take into account the social determinants of health, as they can either help to address existing disparities (eg, sharing relevant information across settings and developing pathways for those most at risk) or inadvertently reinforce them. There are some ongoing initiatives promoting standards to facilitate this (eg, [26]).
**Technological dimensions**		
	Existing infrastructures and legacy systems [27]	A variety of legacy systems exist across sectors, and these may not be set up for wider data sharing across settings.
	Architecture and migration path [28]	There is no validated architecture for delivering integration with no established viable migration paths to deliver desired outcomes.
	Digital maturity: subsectors and organizations [29]	Diverse organizations have different levels of competence in different parts of digitalization and these can vary significantly. The role of digital information and the way it can be shared vary. Therefore, there may in some instances be no data to send, and the data received may have no place to reside in the receiving organization.
	Suppliers [30]	There may be resistance by suppliers to make their systems interoperable (eg, due to commercial interests, technological incapability, lack of resources, and ownership issues).
	Upgrades and maintenance [31]	When one part of the system is upgraded, there may be a risk that other infrastructures cannot cope with this. This is in particular problematic in cases of interfacing between different systems. The rate, direction, and compatibility of evolution may result in failures of the overall infrastructure. Additionally, the infrastructure owner may have significant investment in bespoke software to integrate components and that will require continuous maintenance to adapt to uncoordinated changes in components from different suppliers.
	Hardware and physical infrastructure [32]	There may be connectivity issues, bandwidth problems, and slow internet connection in some areas. There may also be a lack of digital devices, including portable devices and associated apps, which allow frontline staff to connect and retrieve information from the core repository.
	Harmonizing data structures and making systems interoperable [33]	Different systems may be difficult to connect due to incompatible data structures. There are significant costs associated with harmonizing records built around different (earlier) standards. This is particularly true for legacy systems that do not use modern approaches to data.
	Data cleaning and quality management [34]	Data cleaning and data quality management are needed for reuse, but this can be very time-consuming and costly. In addition, the form and quality of data created by one user may not meet the needs of other users. There is also uncertainty about who should be the primary curator, as well as variations in definitions across sectors on what constitutes good-quality data.
	Flows of information [13]	A shared care record depends on multiple sources of information flowing at different speeds through the health and social care system. Inconsistencies due to different timings in updating the information can lead to confusion or mistrust among different users.

Successful examples of shared care have shown that a collaborative and flexible approach with a focus on developing new structures that promote the development of new competencies and ways of working can facilitate mobilization, alignment, and adoption [35]. A technology-driven approach focused on developing administrative procedures, disruptive processes, and top-down decision-making is less likely to be effective.

The highest-priority areas to address and the most important considerations when planning shared care pathways are socio-organizational in nature, as these will determine the suitability and likely adoption of technological solutions. As a first step, the collective needs of stakeholders need to be identified and shared care pathways need to be planned. Technological considerations then need to be considered for addressing identified needs. Achieving the socio-organizational conditions for successful shared care is not an easy task. There are significant power differentials between health and social care organizations. For instance, social care budgets are small by comparison with those of health care, and gaining senior leadership buy-in may be difficult because social services are under severe strain.

## Conclusions

Visions of the digitally supported integration of health and social care have been projected in advance of well-evidenced exemplars of how they might be achieved. The limited understanding of the socio-organizational challenges associated with such transformations has to date resulted in inadequate strategies to tackle emerging tensions.

What is key going forward is the understanding that shared care will involve a transformation of systems, consisting of structures and processes, that go well beyond the confines of individual organizations, and may include at times conflicting agendas. A single architecture is unlikely to fulfill all requirements simultaneously (eg, real-time, dynamic, event-level data centered on the patient and development of stable, curated repositories of longitudinal health records for biomedical research and planning). Hence, there is now a need to identify potential architectural components and designs and map their benefits and trade-offs.

Shared care is difficult but possible. Successful examples have shown that a substantial amount of work is required to mobilize and align stakeholders, often over extended time frames, and plan shared care pathways. Policy makers, planners, and implementers need to work toward achieving and continuously maintaining stakeholder alignment; only in this context can successful technological solutions be developed. Continuous monitoring of the impact of new solutions on the socio-organizational context followed up by re-establishing alignment are essential to achieving shared care.
